# Expressions Profiles of the Proteins Associated with Carbohydrate Metabolism in Rat Liver Regeneration

**DOI:** 10.1155/2017/8428926

**Published:** 2017-07-02

**Authors:** Li Yin, Cuifang Chang, Cunshuan Xu

**Affiliations:** ^1^College of Life Science, Henan Normal University, Xinxiang, Henan Province 453007, China; ^2^State Key Laboratory Cultivation Base for Cell Differentiation Regulation and Henan Bioengineering Key Laboratory, Henan Normal University, Xinxiang, Henan Province 453007, China; ^3^Luohe Medical College, Luohe, Henan Province 462002, China

## Abstract

Liver has a very amazing ability to regenerate from the remnant liver after injury or partial hepatectomy (PH). Carbohydrate metabolism plays a critical role in regeneration. Many signaling pathways are involved in the metabolism process. We analyzed the changes of proteins at 0–36 h after PH in rats using isobaric tags for relative and absolute quantitation (iTRAQ) coupled with LC-MS/MS-based quantitative proteomics strategy. The results showed that 110 proteins and 5 signaling pathways related to carbohydrate metabolism in rat LR changed significantly. Based on a motif discovery method performed by iRegulon, we identified for the first time that the transcription factor SPIB whose motif was enriched among the differentiated genes associated with carbohydrate metabolism may play an important role in liver regeneration for the first time. The findings of this research provide a molecular basis for further unrevealing the mechanism of regeneration at priming stage (0–6 h) and proliferation stage (6–36 h) of LR in rats. At the same time, our studies provide more novel evidence for the signaling pathways which regulate carbohydrate metabolism from proteomics level. This study can provide some new thinking of liver regeneration and treatment of diseases associated with glucose metabolism.

## 1. Introduction

Liver has a very amazing ability to regenerate. The remnant liver can enter the cell cycle rapidly after encountering some stimulus such as injury or partial hepatectomy (PH) in order to substitute the lost hepatic tissue and restore liver function. This process is called liver regeneration (LR) [1]. Under normal circumstances, the majority of liver cells are in G0 phase and only a handful of them divide [2]. When a number of factors such as viruses, autoimmune diseases, toxins, drugs, and surgical resection cause liver damage, the usually quiescent and highly terminal differentiated liver cells enter into G1 phase from G0 phase after receiving the signal stimulation and then induce the expression of a series of genes related to liver regeneration [3]. The proliferation of the hepatocytes completes twice in 2-3 days and is accompanied by the proliferation of hepatic stellate cells, Kupffer cells, and biliary epithelial cells. At the same time, the proliferation of endothelial cells and angiogenesis are involved in the reconstruction of liver construction too [4].

It is generally believed that LR is divided into three stages, priming, proliferation, and termination, and the specific time is species-dependent. In rat, the priming stage lasts for 4 h which characterized the transition from G0 to the cell cycle and DNA synthesis initiates approximately 20 h after hepatectomy [5]. Cell activation, proliferation, and apoptosis and other physiological activities in LR are closely associated with the normal liver function, development, growth, and disease [6–8]. Thus, the illumination of the molecular mechanism of LR has an important theoretical and practical value to reveal the mechanism of liver disease and establish the methods of treatment and prevention of liver disease [9–11]. As the center of metabolism, liver plays an important role in glucose utilization, absorption, transport, and degradation. It maintains a relatively constant blood glucose level mainly by glycogen synthesis, glycolysis, glycogenolysis, and gluconeogenesis. Glycogen can be broken down into glucose and released into the blood to regulate the normal blood sugar levels but also can be converted into a variety of polysaccharides, oligosaccharides, and glucose derivatives so as to constitute the cell structure [12].

The carbohydrate metabolism activity which occurs in liver contains mainly glycolysis, aerobic oxidation, pentose phosphate pathway, glycogen synthesis, glycogenolysis, and gluconeogenesis. Glucose, fructose, and galactose can enter the glycolytic pathway through phosphorylation of various forms so as to perform glycolysis or aerobic oxidation so as to provide ATP which the body needs. In this study, the glucose metabolism proteomics in rat liver regeneration were studied by means of high-throughput biological analysis and systems biology approach. We found that six signaling pathways and many kinds of proteins regulate the carbohydrate metabolism after combination of the databases from NCBI, GENEONTOLOGY, RGD, KEGG, and IPA (Ingenuity Pathway Analysis) software. We expanded the study at translational level in order to explore the activity and mechanism of glucose metabolism-related physiological process of LR. In this study, the protein expression profile was examined which is related to rat LR using isotopically labeled tags for relative and absolute quantification (iTRAQ) combined with mass spectrometry (MS) [13–17]. And we analyzed the likely transcription factor which is likely regulating the carbohydrate metabolism and built the network.

## 2. Materials and Methods

### 2.1. Model Preparations of Rat Liver Regeneration Induced by Partial Hepatectomy

All rats in our study were obtained from the Experimental Animal Center of Henan Normal University, and protocols were approved by Institutional Animal Care and Use Committee of Henan Normal University in China (Permit Number SYXK2008-0105). Adult healthy male Sprague-Dawley rats weighing 210 g–250 g were provided by the Experimental Animal Center of Henan Normal University. These rats were raised in a controlled temperature room (21 ± 2°C) with the relative humidity 60 ± 10% and illumination 12 h/d (light from 8:00 to 20:00). They were allowed to have water and food freely. A total of 76 rats were divided into 19 groups with 4 rats per group: one normal control group (NC), nine sham-operated groups (SO), and nine partial hepatectomy groups (PH). The rats in PH groups were subjected to 2/3 partial hepatectomy according to the method described previously (Xu et al., 2010). The SO group received the same procedure as the PH group except the liver removal and the same procedure as the PH group. The rats were anesthetized and put to death at 0, 2, 6, 12, 24, 30, and 36 h after operation according to Higgins' methods; that is to say, the middle and the left hepatic lobe accounting for about 70% of liver weight were removed. The experimental procedures were in accordance with the Current Animal Protection Law of China.

### 2.2. Protein Extractions, iTRAQ Labeling, and Peptides Isolation

Protein extraction was performed according to the procedure described before [18]. Frozen rat liver samples were grounded to powder in liquid nitrogen and suspended in lysis buffer (150 mM Tris, 8 M urea, 2 M thiourea, 4% CHAPS, and 65 mM DTT). The suspension was vortexed at 4°C for 1 h and then centrifuged at 20,000*g* for 1 h in a high-speed centrifuge. The protein concentration of each sample was determined by 2D Quantification kit (GE Healthcare, USA).

A total of 80 *μ*g of each sample was denatured, reduced, and alkylated as described in the iTRAQ protocol (Applied Biosystems, USA). Each sample was digested with 0.1 *μ*g/*μ*L trypsin solution at 37°C and overnight. Control (0 h), 2 h, 6 h, 12 h, 24 h, 30 h, and 36 h samples were, respectively, labeled with 114, 115, 116, 117, 118, 119, and 121 iTRAQ tags according to the manufacturer's protocol (Applied Biosystems, USA). Seven samples were collected and vacuum-dried. All labeled peptides were mixed to be performed by SCX prefraction. The flow-through and elution were collected in 30 parts and merged into eight samples per group according to SCX chromatogram. The samples were desalted using a Pepclean C^18^ spin column (sigma, USA) and dried by vacuum centrifugation. Each sample was analyzed by mass spectrometry (Thermo Fisher Scientific, Waltham, MA, USA) after the capillary HPLC separation [2, 15].

The style of the original data got from mass spectrometry is RAW. We uploaded the RAW data to Mascot 2.2 software (Matrix Science, London, UK) by Proteome Discover 1.4 and searched through the Swiss-Prot database from Uniprot website (http://www.uniprot.org). The analysis and search parameters were set as described before [13].

### 2.3. The Validation of iTRAQ Data by Western Blot Validation

The Western blot was performed as described previously to verify the expression of proteins. In brief, the proteins were separated and transferred to a PVDF membrane. Then, the membrane was blocked in Tris-buffered saline containing 0.1% tween-20 for 1 h at 37°C. Subsequently, the membrane was incubated with primary antibodies rabbit anti-CD68 (Boster, #BA3638, 1 : 1,000), rabbit anti-CD163 (Bioss, bs-2527R, 1 : 1,000), rabbit anti-CD47 (Boster, # BM413, 1 : 1,000), and rabbit anti-PIK3R1 (Boster, # BA1352-2, 1 : 1,000) and then the secondary antibodies (alkaline phosphatase labeled) for 1 h at 37°C. Finally, the membrane was visualized in the substrate solution and terminated by TE buffer. The relative quantity of target proteins was analyzed by Typhoon 9500 and ImageQuant software with *β*-actin as the internal reference.

### 2.4. Master Regulator Analysis

In order to identify TFs based on regulatory motif and chromatin immunoprecipitation-sequencing, we performed iRegulon plugin in Cytoscape v3.3.4 [19]. The master regulator and its targets were detected by scanning the known TF-binding promoter motifs. All the options were taken as default.

### 2.5. The Dysregulation Prediction of Signaling Pathways Participating in the Carbohydrate Metabolism

In order to find the changes of signaling pathways associated with glucose metabolism, we used the Ingenuity Pathway Analysis (IPA) to perform comparative analysis. Briefly, the dataset (.txt style) containing the expression value of our proteins was uploaded to IPA. Then the core analysis and comparison analysis were performed to get the heat map of every signaling pathway and carbohydrate metabolism.

## 3. Results

### 3.1. The Expression of Proteins Related to Carbohydrate Metabolism in Rat Liver Regeneration

According to protein expression profile related to carbohydrate metabolism detected by iTRAQ, we found that 110 kinds of protein were associated with liver regeneration, wherein PKA, PPAR, P53, HIF1*α*, PIK3/AKT, and AMPK signaling pathway and glucose metabolism-related proteins have 27, 16, 18, 7, 35, 12, and 33 kinds, respectively, as shown in Supplementary Table  1 in Supplementary Material available online at https://doi.org/10.1155/2017/8428926.

### 3.2. Cluster of the Protein Expression Patterns Related to Carbohydrate Metabolism and the Signaling Pathways Participating in Carbohydrate Metabolism in Rat LR and the Validity of Western Blot Validation

**Figure 1:**
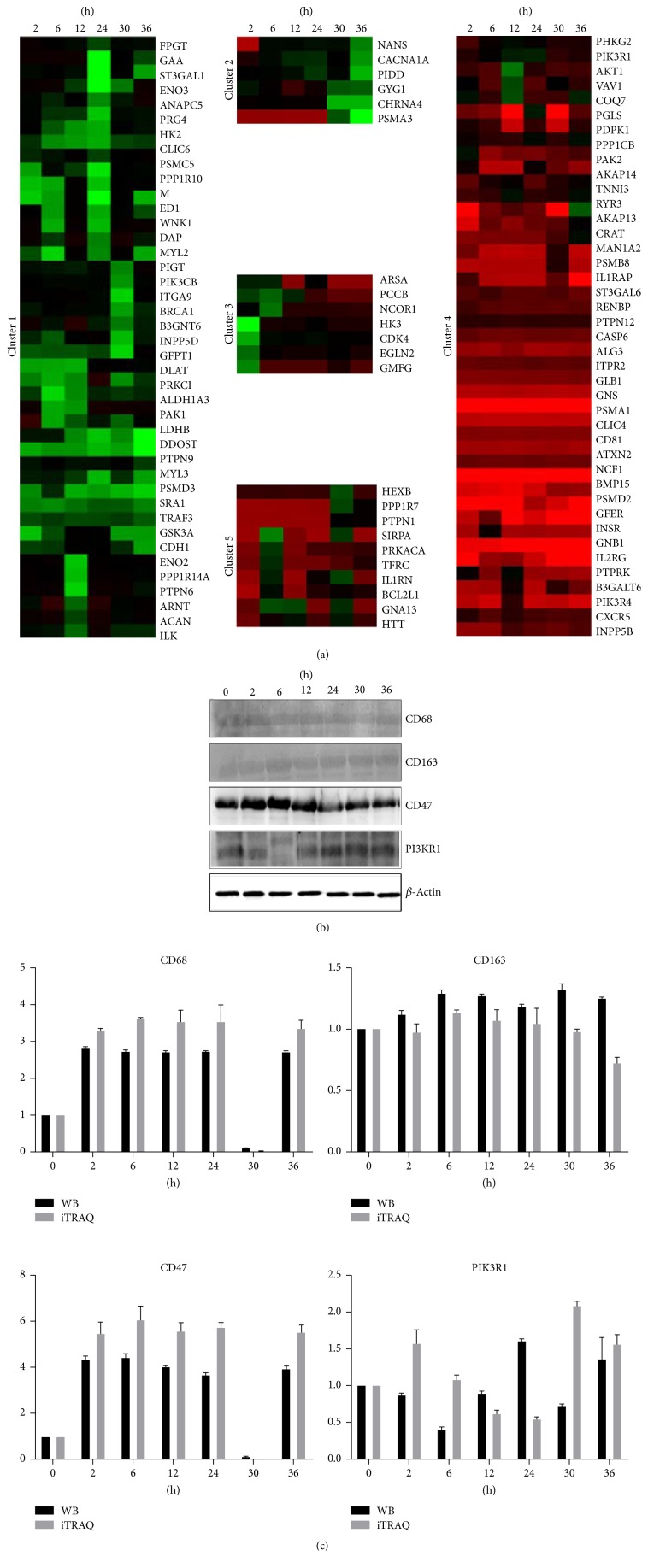
Global protein expression patterns in rat LR and Western blot validation. (a) The “hierarchical diagram” column represents 109 differentially expressed proteins. Red and green colors represent the expression level higher and lower than the control, respectively. (b) The protein expression levels detected by Western blot. *β*-Actin is the internal reference. (c) The correlation of proteins detected by iTRAQ and Western blot. The horizontal axis represents the recovery time (h) after partial hepatectomy and the ordinate axis represents the relative protein level.

Cluster 1 was mainly enriched with the proteins which were downregulated at almost all time points including CLIC6, SPA1, PSMD3, and DDOST. The proteins in cluster 2 were classified together mainly because they were downregulated at the proliferation stage and changed slightly, even a little, at other time points including NANS, CACNA1A, and GYG1. The third cluster mainly included proteins downregulated at priming stage and upregulated at the proliferation stage such as ARSA, PPP1CB, NCOR1, HK3, and CDK4. Almost all the proteins that were upregulated at all time points are classified into the fourth cluster including GLB1, AKT1, COQ7, INSR, and PIK3R1. The last cluster included mainly the proteins that were up-/downregulated including HEXB, PPP1R7, PTPN1, and SIRPA (Figure 1(a)).

To validate the reliability of the iTRAQ results, 4 proteins were detected by Western blot detection including CD68, CD163, CD47, and PIK3R1 (Figure 1(b)). The results showed that there is a good correlation in the expression level of 4 proteins between Western blot and iTRAQ result (Figure 1(c)).

### 3.3. Master Regulators Analysis

We discovered the regulatory TFs at the initiation of LR using iRegulon in Cytoscape. The most strongly enriched TF motif is SPIB with a normalized enrichment score (NES) of 5.299. The expression changes of the target genes for SPIB have been shown in color in Figure 3.

### 3.4. The Expression of Proteins Associated with Carbohydrate Metabolism Signaling Pathways and the Predicted Signaling Activity in LR

**Figure 2:**
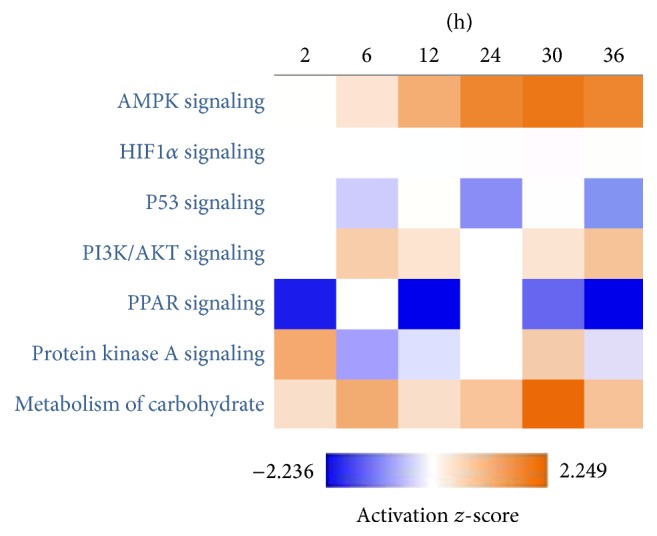
The comparison analysis of canonical pathway associated with carbohydrate metabolism. Orange represents enhancement and blue represents decrease.

**Figure 3:**
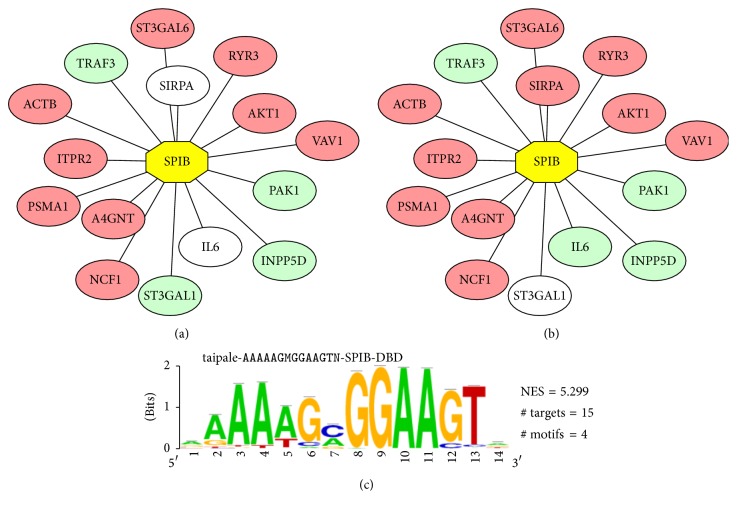
Network of SPIB using iRegulon in Cytoscape. The nodes in pink and green represent the upregulated and downregulated genes at the initiation and progression stage of LR in rat. (a) The regulator SPIB and its targets genes at the initiation stage of LR. (b) The regulator SPIB and its target genes at the progression stage of LR. (c) The binding motif of SPIB.

The protein expression profile of the signaling pathways associated with LR was uploaded to IPA to perform the Heatmap analysis by the Ingenuity® Knowledge Base database built in IPA. The result indicated that four out of five signaling pathways changed significantly, one activated, two suppressed, and one changed differently at different time points. The activity of AMPK signaling pathway increased, p53 signaling pathway decreased at 6 h, 24 h, and 36 h after hepatectomy, and PPAR signaling pathway weakened at 2 h, 30 h, and 36 h as shown in Figure 2. Notably, HIF1*α* signaling did not change. And the carbohydrate metabolism was upregulated at all time points and changed significantly, except at 12 h.

## 4. Discussion

**Figure 4:**
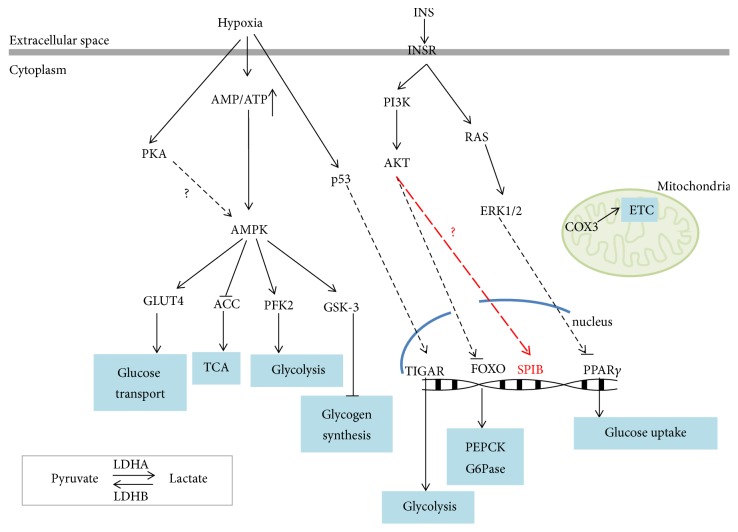
The crosstalk between carbohydrate metabolism and the signaling pathways.

Adenosine monophosphate activated protein kinase (AMPK) signaling pathway plays an important role in the energy metabolism, especially in carbohydrate metabolism [20–23]. AMPK consists of three kinds of subunits: *α*, *β*, and *γ*. There are 12 different existing forms and the main form in rat liver is *α*1/*β*1/*γ*1 and *α*2/*β*1/*γ*1 [24–27]. AMPK can be activated by an increased ratio of AMP/ATP or ADP/ATP [28]. Studies have shown that activation of AMPK inhibits glycogen synthase, activates glucose transporter 4 (GLU4), and then facilitates the transfer and absorption of glucose and increases the glycogen content in skeletal muscle [29]. AMPK is very sensitive to hypoxia. The AMP/ATP and creatine/phosphocreatine ratio influence AMPK signaling pathway significantly. Our research found that AMPK signaling pathways and carbohydrate metabolism have been in active state almost in the whole process of LR as shown in Figure 4. In order to illustrate clearly the changes of the proteins in rat LR, we divided the liver regeneration into two stages, initiation stage (2–6 h) and proliferation stage (6 h–36 h). After PH, the low nutrients caused the rise of AMP/ATP ratio and activated PKA, and the latter activated the STK11-STRAD-MO25 ternary complex and caused the phosphorylation of *α* subunit in AMPK. When *α* subunit combines with *β* and *γ* subunit, AMPK gets activated. The activated AMPK caused four effects at least from our results as follows. Firstly, it activated GLUT4 so as to increase glucose transport and strengthen glucose utilization. Secondly, AMPK induced phosphorylation of ACC, thereby inhibiting its function and the synthesis of malonyl-CoA and fatty acid from acetyl-CoA, so that the latter entered into the Krebs cycle to produce more ATP which can meet the energy needs of regeneration. Thirdly, AMPK caused the phosphorylation of PFK-2, which catalyzed generation of F-2 and 6-P2 and the latter produced F-1 and 6-P2 under the upregulated PFK-1. Pyruvate was eventually produced and entered into the TCA cycle to generate ATP. Fourthly, GSK3 forms complex with AMPK *β*-regulatory subunit and phosphorylates AMPK*α*-catalytic subunit [30]. Based on previous researches, the activity of GSK-3 was reduced when its ser9 site was phosphorylated by AKT [31]. And GSK-3 phosphorylates glycogen synthase, thus inhibiting its activity.

Our research found that the expression of the insulin receptor which resides in the membrane was upregulated. The activated insulin receptor causes the phosphorylation of tyrosine of IRS 1,2. The activated IRS activated PI3K and the latter caused the phosphorylation of phosphatidylinositol 4,5-bisphosphate (PIP2) to produce PIP3. As the second messenger, PIP3 can activate AKT and the latter inhibits FOXO. As the first clarified kinase that inhibits FOXO, AKT played an extremely important role in the regulation of FOXO [32, 33]. The activated AKT promoted the phosphorylation of FOXO and caused its inactivity. The inactivated FOXO was transported from nucleus to the cytoplasm, thereby terminating the action on G6Pase, FBPase, and PEPCK and inhibited glucose production at the level of transcription. In our study, FBPase was upregulated at the priming and proliferation stage, which agreed with previous studies [34, 35]. The deletion of p53 caused the reduction of oxygen consumption and resulted in the attenuation of aerobic oxidation [36]. In order to ensure the energy demand, glycolysis strengthened. So p53 is the very important substance in balancing the glucose metabolism and aerobic oxidation. p53 regulates the aerobic respiration process in mitochondria mainly depending on the regulation of regulative subunit of cytochrome c oxidase complex. Cytochrome c oxidase (COX) catalyzes the transfer of electrons from cytochrome C to molecular oxygen, which helps to maintain the electrochemical gradient between outer and inner mitochondrial membrane that is necessary to synthesize ATP. In our study, LDHA (M type lactate dehydrogenase) has been in a rising state while LDHB (H type lactate dehydrogenase) in a reduced state in the whole process of LR. As we all know, LDHA has a relatively lower *K*_*m*_ value to pyruvate and promotes the transformation of pyruvate to lactate easily. And the following glycolysis could provide energy quickly relatively. LDHB has a higher *K*_*m*_ to lactate; what it does more is promoting the transformation from lactate to pyruvate and the latter goes into Krebs cycle. In order to compensate for the lack of energy, the glycolysis goes on for a longer time. That is to say, glycolysis makes up for the reduction of energy due to the reduction of aerobic oxidation [37]. The result is consistent with Matoba et al.'s [38].

PPARs are nuclear hormone receptor superfamily members including 3 kinds of subtypes, PPAR*α*, PPAR*δ*, and PPAR*γ* [39], and PPAR*α* mainly is in liver. It is reported that insulin inhibited the expression of PPAR*α* which is highly expressed in liver [40]. PPAR*α* regulated lipid metabolism by regulating some enzymes participating in *β*-oxidation in peroxisome [41]. According to Zeng et al.'s report, the drugs acting on PPAR*γ* affected the process of glucose metabolism by affecting the activity of key enzyme in glycolysis [42]. And, according to Yan's report, the Geniposide's hypoglycemic effects were directly related to the activation of PPAR*γ* receptor (Yan et al. 2007). In our study, PPAR signaling pathway has been in a suppressed state. Therefore, we speculate that a lot of energy is required at priming and proliferation stage of LR, but the *β*-oxidation of fatty acids is blocked and more glucose is employed due to the lack of oxygen.

HIF is a major transcription factor regulating transcription of most enzymes in the glycolytic pathway from glucose down to lactate [36]. In our study, HIF1*α* signaling pathway did not change significantly. But the downstream molecule of the signaling pathway such as PDK1 changed and thereby regulated the glucose metabolism in LR. Hypoxia inducible factor (HIF) is the most important factor which maintains the oxygen balance in mammals with pyruvate dehydrogenase kinase-1 (PDK1) as an important target gene. The protein encoded by PDK1 has a direct impact on the fate of pyruvate. Our research found that PDK1 declined slightly at priming stage and increased at proliferation stage which is consistent with the SO group, so PDK1 is related to liver injury (not shown). Based on the above process, PDK1 inhibited the synthesis of acetyl coenzyme A by phosphorylating PDH, thus blocking Krebs cycle and reducing oxygen consumption. At the same time, the increased LDHA promoted more pyruvate to generate lactate. This was consistent with Simon [43] (Figure 4).

The relatively new transcription factor SPIB belongs to ETS family and binds to purine-rich sequence and plays an important role in differentiation [44]. SPIB is overexpressed in several cancers including liver and colon cancers compared to the normal samples. And the high SPIB was significantly associated with the poor survival of patients with HCC so it may serve as a clinical prognostic indicator of HCC [45]. In our study, the target genes regulated by SPIB are overexpressed the most. Considering the similarity between regeneration and tumor, we inferred that SPIB may play a vital role in liver regeneration.

In conclusion, the carbohydrate metabolism is activated at the priming and progression stage during LR. At priming stage, the activation of metabolism may be prepared for the requirement of substance and energy for the ensuring cell cycle. Due to the deficiency of oxygen at the priming stage, the glycolysis may play a more important role. At the progression stage, more energy is required for the synthesis of DNA and proteins and the carbohydrate is more metabolic than the priming stage. As the most important organ in regulating relatively constant blood sugar levels, liver regulates glucose metabolism by many metabolic pathways. At the priming stage of live regeneration, the most important thing is to reduce the oxygen consumption and increase glycolysis to provide energy. Many signaling pathways and proteins related to glucose metabolism intercross with each other in maintaining the balance of energy and blood sugar. At the priming stage, glycolysis played an important role in the energy supply due to the deficiency of oxygen. At the progressing stage, aerobic oxidation supplied more energy for cell proliferation. And we identified that the transcription factor SPIB may play a significant role in liver regeneration.

## Supplementary Material

Supplementary Table 1 the expression of proteins sugar related to metabolism in rat liver regeneration detected by iTRAQ.
