# Supplementary material for: Expressions Profiles of the Proteins Associated with Carbohydrate Metabolism in Rat Liver Regeneration
Source: Biomed Res Int. 2017 Jul 2;2017:8428926. doi: 10.1155/2017/8428926 (PMC5511655; doi:10.1155/2017/8428926)
Supplement: Supplementary file 1 — Supplementary Table 1 the expression of proteins sugar related to metabolism in rat liver regeneration detected by iTRAQ. [file 8428926.f1.pdf]

Supplementary table 1 the expression of proteins sugar related to metabolism in rat liver regeneration detected by iTRAQ

| Protein name | Recovery time points (h) after PH | | | | | | |
|---|---|---|---|---|---|---|---|
| | 0h | 2h | 6h | 12h | 24h | 30h | 36h |
| **A. Signaling pathways** | | | | | | | |
| **a. PKA Signaling** | | | | | | | |
| GNB1 | 1.00 | 53.58 | 54.12 | 77.81 | 94.94 | 919.51 | 991.02 |
| CLIC4 | 1.00 | 22.82 | 25.10 | 25.59 | 24.72 | 24.07 | 24.74 |
| ITPR2 | 1.00 | 5.52 | 4.82 | 4.99 | 4.38 | 5.04 | 4.53 |
| PTPN12 | 1.00 | 2.08 | 2.02 | 2.35 | 2.12 | 2.19 | 2.17 |
| AKAP13 | 1.00 | 40.39 | 5.26 | 10.37 | 5.57 | 4.26 | 1.05 |
| PTPN1 | 1.00 | 12.11 | 12.06 | 12.21 | 12.44 | 1.01 | 1.13 |
| PPP1CB | 1.00 | 0.76 | 10.94 | 6.33 | 7.24 | 4.52 | 3.22 |
| PTPRK | 1.00 | 1.20 | 7.67 | 0.88 | 12.17 | 12.87 | 14.88 |
| TNNI3 | 1.00 | 2.90 | 2.20 | 0.77 | 2.09 | 1.77 | 0.83 |
| AKAP14 | 1.00 | 4.78 | 1.59 | 1.14 | 4.40 | 1.75 | 1.17 |
| PHKG2 | 1.00 | 3.29 | 1.23 | 0.83 | 1.09 | 1.67 | 1.63 |
| CLIC6 | 1.00 | 0.38 | 0.31 | 0.05 | 0.07 | 0.10 | 0.46 |
| MYL3 | 1.00 | 0.07 | 0.46 | 0.05 | 0.38 | 0.69 | 0.64 |
| GSK3A | 1.00 | 0.07 | 0.07 | 0.95 | 1.06 | 0.28 | 0.04 |
| PPP1R10 | 1.00 | 0.29 | 0.94 | 0.06 | 1.08 | 0.07 | 1.04 |
| CACNA1A | 1.00 | 1.62 | 0.86 | 0.53 | 0.49 | 1.01 | 0.08 |
| PTPN9 | 1.00 | 0.37 | 1.00 | 0.77 | 0.75 | 0.96 | 0.73 |
| MYL2 | 1.00 | 1.15 | 0.11 | 0.90 | 0.60 | 0.71 | 0.79 |
| PHKB | 1.00 | 1.08 | 1.01 | 0.33 | 1.03 | 0.76 | 0.52 |
| ANAPC5 | 1.00 | 1.36 | 0.69 | 0.10 | 1.05 | 0.57 | 0.91 |
| PPP1R14A | 1.00 | 0.94 | 1.09 | 1.11 | 0.04 | 0.97 | 1.09 |
| PTPN6 | 1.00 | 0.76 | 1.19 | 1.24 | 0.03 | 1.15 | 1.14 |
| RYR3 | 1.00 | 93.07 | 6.78 | 3.54 | 5.76 | 82.23 | 0.22 |
| PRKACA | 1.00 | 7.58 | 0.41 | 7.59 | 3.04 | 2.92 | 2.33 |
| PPP1R7 | 1.00 | 12.29 | 12.12 | 11.88 | 11.40 | 0.32 | 1.12 |
| SIRPA | 1.00 | 13.15 | 0.12 | 8.01 | 13.59 | 0.39 | 11.35 |
| GNA13 | 1.00 | 4.95 | 0.28 | 0.32 | 7.11 | 0.48 | 7.17 |
| **b.PPAR Signaling** | | | | | | | |
| BMP15 | 1.00 | 36.70 | 46.90 | 57.66 | 32.92 | 11.49 | 12.42 |
| GFER | 1.00 | 24.37 | 5.10 | 556.00 | 37.29 | 560.26 | 553.54 |
| PIK3R4 | 1.00 | 20.29 | 32.04 | 2.10 | 27.49 | 22.85 | 34.79 |
| IL1RAP | 1.00 | 2.97 | 18.62 | 20.26 | 20.72 | 4.56 | 79.78 |
| INSR | 1.00 | 15.47 | 1.23 | 15.33 | 13.88 | 15.46 | 15.79 |
| PIK3R1 | 1.00 | 1.77 | 1.08 | 0.61 | 0.58 | 2.15 | 1.69 |
| SRA1 | 1.00 | 0.11 | 0.22 | 0.23 | 0.15 | 0.10 | 0.12 |
| IL6 | 1.00 | 0.96 | 0.98 | 0.04 | 1.08 | 0.06 | 0.04 |
| MED1 | 1.00 | 1.15 | 1.12 | 0.04 | 1.17 | 0.05 | 1.18 |
| PIK3CB | 1.00 | 1.03 | 0.03 | 0.88 | 1.07 | 1.05 | 1.08 |
| ITGA9 | 1.00 | 0.55 | 0.07 | 0.89 | 0.90 | 0.98 | 0.71 |
| PRKACA | 1.00 | 7.58 | 0.41 | 7.59 | 3.04 | 2.92 | 2.33 |
| GMFG | 1.00 | 0.11 | 3.46 | 3.33 | 3.46 | 1.64 | 3.56 |
| IL1RN | 1.00 | 12.05 | 0.60 | 11.73 | 1.21 | 0.33 | 6.19 |
| AKT1 | 1.00 | 1.02 | 3.95 | 0.30 | 3.92 | 1.03 | 2.13 |
| NCOR1 | 1.00 | 0.94 | 0.14 | 1.84 | 1.84 | 1.94 | 2.12 |
| **c.p53 Signaling** | | | | | | | |
| PSMD2 | 1.00 | 536.75 | 553.64 | 555.59 | 15.97 | 35.90 | 487.43 |
| PSMA1 | 1.00 | 213.96 | 202.78 | 204.83 | 212.72 | 214.45 | 214.03 |
| BMP15 | 1.00 | 36.70 | 46.90 | 57.66 | 32.92 | 11.49 | 12.42 |
| GFER | 1.00 | 24.37 | 5.10 | 556.00 | 37.29 | 560.26 | 553.54 |
| PIK3R4 | 1.00 | 20.29 | 32.04 | 2.10 | 27.49 | 22.85 | 34.79 |
| CASP6 | 1.00 | 4.73 | 6.30 | 6.52 | 5.73 | 5.54 | 6.17 |
| PSMB8 | 1.00 | 19.18 | 18.93 | 18.69 | 18.75 | 1.80 | 18.22 |
| PIK3R1 | 1.00 | 1.77 | 1.08 | 0.61 | 0.58 | 2.15 | 1.69 |
| PSMD3 | 1.00 | 0.03 | 0.05 | 0.08 | 0.06 | 0.51 | 0.10 |
| PSMC5 | 1.00 | 0.05 | 1.00 | 0.01 | 1.03 | 0.07 | 0.03 |
| CDK4 | 1.00 | 0.06 | 0.86 | 0.94 | 0.83 | 1.06 | 1.09 |
| BRCA1 | 1.00 | 0.62 | 0.13 | 0.63 | 1.87 | 0.76 | 1.30 |
| PIK3CB | 1.00 | 1.03 | 0.03 | 0.88 | 1.07 | 1.05 | 1.08 |
| IL6 | 1.00 | 0.96 | 0.98 | 0.04 | 1.08 | 0.06 | 0.04 |
| PIDD | 1.00 | 1.03 | 0.94 | 0.79 | 0.40 | 0.92 | 0.05 |

| Protein name | Recovery time points (h) after PH | | | | | | |
|---|---|---|---|---|---|---|---|
| | 0h | 2h | 6h | 12h | 24h | 30h | 36h |
| PSMA3 | 1.00 | 11.30 | 11.10 | 11.55 | 11.68 | 0.25 | 0.01 |
| GMFG | 1.00 | 0.11 | 3.46 | 3.33 | 3.46 | 1.64 | 3.56 |
| AKT1 | 1.00 | 1.02 | 3.95 | 0.30 | 3.92 | 1.03 | 2.13 |
| **d.HIF1α signaling** | | | | | | | |
| PIK3R4 | 1.00 | 20.29 | 32.04 | 2.10 | 27.49 | 22.85 | 34.79 |
| PIK3R1 | 1.00 | 1.77 | 1.08 | 0.61 | 0.58 | 2.15 | 1.69 |
| LDHB | 1.00 | 0.00 | 0.10 | 0.04 | 0.10 | 0.71 | 0.72 |
| EGLN2 | 1.00 | 0.17 | 1.13 | 1.37 | 1.35 | 1.03 | 1.52 |
| PIK3CB | 1.00 | 1.03 | 0.03 | 0.88 | 1.07 | 1.05 | 1.08 |
| AKT1 | 1.00 | 1.02 | 3.95 | 0.30 | 3.92 | 1.03 | 2.13 |
| ARNT | 1.00 | 1.08 | 0.93 | 1.80 | 0.18 | 2.18 | 0.82 |
| **e.HER-2 signaling** | | | | | | | |
| GSK3A | 1.00 | 0.07 | 0.07 | 0.95 | 1.06 | 0.28 | 0.04 |
| PRKCI | 1.00 | 0.54 | 0.39 | 0.56 | 0.10 | 0.03 | 0.39 |
| AKT1 | 1.00 | 1.02 | 3.95 | 0.30 | 3.92 | 1.03 | 2.13 |
| **f.AMPK Signaling** | | | | | | | |
| GNB1 | 1.00 | 53.58 | 54.12 | 77.81 | 94.94 | 919.51 | 991.02 |
| PIK3R4 | 1.00 | 20.29 | 32.04 | 2.10 | 27.49 | 22.85 | 34.79 |
| CRAT | 1.00 | 8.98 | 7.91 | 7.78 | 7.59 | 3.09 | 0.81 |
| INSR | 1.00 | 15.47 | 1.23 | 15.33 | 13.88 | 15.46 | 15.79 |
| PDPK1 | 1.00 | 1.23 | 2.02 | 2.14 | 0.87 | 2.10 | 0.81 |
| PIK3R1 | 1.00 | 1.77 | 1.08 | 0.61 | 0.58 | 2.15 | 1.69 |
| CHRNA4 | 1.00 | 1.13 | 1.06 | 1.13 | 1.13 | 0.05 | 0.05 |
| PIK3CB | 1.00 | 1.03 | 0.03 | 0.88 | 1.07 | 1.05 | 1.08 |
| PRKACA | 1.00 | 7.58 | 0.41 | 7.59 | 3.04 | 2.92 | 2.33 |
| TFRC | 1.00 | 6.42 | 0.50 | 6.55 | 6.55 | 3.73 | 1.76 |
| GNA13 | 1.00 | 4.95 | 0.28 | 0.32 | 7.11 | 0.48 | 7.17 |
| AKT1 | 1.00 | 1.02 | 3.95 | 0.30 | 3.92 | 1.03 | 2.13 |
| **B.carbohydrate metabolism** | | | | | | | |
| GNS | 1.00 | 20.04 | 19.99 | 18.24 | 19.83 | 21.40 | 19.88 |
| ALG3 | 1.00 | 11.59 | 13.36 | 9.51 | 14.97 | 14.28 | 12.13 |
| GLB1 | 1.00 | 8.54 | 8.46 | 8.10 | 8.02 | 7.94 | 7.17 |
| ST3GAL6 | 1.00 | 4.02 | 2.76 | 4.36 | 4.31 | 3.56 | 5.69 |
| RENBP | 1.00 | 2.87 | 4.20 | 4.77 | 4.50 | 4.72 | 4.26 |
| B3GALT6 | 1.00 | 16.67 | 15.07 | 1.36 | 20.23 | 2.04 | 5.83 |
| COQ7 | 1.00 | 5.68 | 8.52 | 157.98 | 0.65 | 173.06 | 4.07 |
| MAN1A2 | 1.00 | 6.48 | 20.87 | 24.57 | 21.16 | 1.85 | 24.73 |
| PPP1CB | 1.00 | 0.76 | 10.94 | 6.33 | 7.24 | 4.52 | 3.22 |
| PGLS | 1.00 | 1.53 | 2.52 | 29.07 | 2.30 | 28.37 | 1.96 |
| ARSA | 1.00 | 0.61 | 0.58 | 9.57 | 1.01 | 9.90 | 10.07 |
| PHKG2 | 1.00 | 3.29 | 1.23 | 0.83 | 1.09 | 1.67 | 1.63 |
| DDOST | 1.00 | 0.00 | 0.07 | 0.05 | 0.08 | 0.08 | 0.06 |
| DLAT | 1.00 | 0.43 | 0.09 | 1.81 | 0.10 | 0.06 | 0.12 |
| LDHB | 1.00 | 0.00 | 0.10 | 0.04 | 0.10 | 0.71 | 0.72 |
| ENO3 | 1.00 | 1.31 | 0.13 | 0.01 | 0.37 | 1.22 | 1.15 |
| ALG11 | 1.00 | 0.91 | 0.98 | 0.02 | 0.37 | 0.85 | 0.07 |
| HK2 | 1.00 | 0.77 | 0.97 | 0.06 | 0.09 | 0.24 | 1.00 |
| GFPT1 | 1.00 | 0.93 | 0.99 | 0.98 | 0.06 | 0.06 | 0.07 |
| GAA | 1.00 | 0.31 | 1.01 | 0.01 | 1.27 | 1.29 | 1.25 |
| ST3GAL1 | 1.00 | 0.07 | 0.98 | 0.00 | 0.80 | 0.98 | 0.87 |
| B3GNT6 | 1.00 | 0.68 | 0.04 | 1.08 | 0.99 | 0.23 | 0.56 |
| ALDH1A3 | 1.00 | 1.26 | 1.36 | 1.45 | 0.08 | 0.06 | 1.27 |
| HK3 | 1.00 | 0.01 | 1.49 | 1.37 | 1.04 | 1.56 | 1.58 |
| PIGT | 1.00 | 0.92 | 0.08 | 0.93 | 0.90 | 0.88 | 0.99 |
| FPGT | 1.00 | 0.84 | 0.91 | 0.31 | 0.85 | 1.04 | 1.23 |
| PHKB | 1.00 | 1.08 | 1.01 | 0.33 | 1.03 | 0.76 | 0.52 |
| ENO2 | 1.00 | 1.06 | 0.98 | 1.06 | 0.04 | 1.12 | 1.05 |
| HEXB | 1.00 | 2.41 | 2.32 | 2.58 | 2.40 | 0.28 | 2.70 |
| A4GNT | 1.00 | 2.49 | 3.76 | 0.14 | 1.86 | 3.22 | 2.82 |
| PCCB | 1.00 | 0.37 | 0.19 | 0.55 | 2.55 | 4.60 | 4.45 |
| NANS | 1.00 | 18.67 | 0.87 | 0.89 | 0.78 | 0.80 | 0.11 |
| GYG1 | 1.00 | 0.72 | 1.10 | 2.63 | 1.32 | 0.25 | 0.21 |

* The red bottom represent up-regulated (>2 fold) proteins, green bottom represent down-regulated (<0.5 fold)proteins
